# Supplementary material for: Efficacy and durability of bovine virus diarrhea (BVD) virus killed vaccine adjuvanted with monolaurin
Source: PLoS One. 2022 Jul 14;17(7):e0269031. doi: 10.1371/journal.pone.0269031 (PMC9282602; doi:10.1371/journal.pone.0269031)
Supplement: S1 File — (DOCX) [file pone.0269031.s001.docx]

**Preparation of monolaurin**

The enzymatic production of glycerin monolaurate (GML) was done via the lipase-catalyzed esterification of lauric acid and glycerin in a solvent-free medium according to a previously described technique [18]. The enzymatic production of glycerin monolaurate (GML) was carried out by lipase catalysed esterification of lauric acid and glycerin in a solvent-free media. Response surface methodology (RSM), based on 5-level and 3-variable composite design, was employed to study the interactive effects of reaction temperature (48–60 °C), enzyme load (1–4% w/w), and glycerin to lauric acid molar ratio (1:1–4:1) on glycerin laurate yield. The optimum conditions obtained were a temperature of 60 °C, an enzyme load of 4%, and a glycerin to lauric acid molar ratio of 4:1. The maximum predicted, and experimental conversion values were 92.26% and 93.23%, respectively. Utilisation of Lipozyme RM IM (Rhizomucor miehei lipase) allowed the formation of a mixture consisting of 50% monoglyceride, 34.6% diglyceride and 8.4% triglyceride, which fulfills the requirements established by the World Health Organization (WHO) for use as a food emulsifier. In addition, the Lipozyme RM IM maintained more than 90% of its original activity after being used for six cycles.


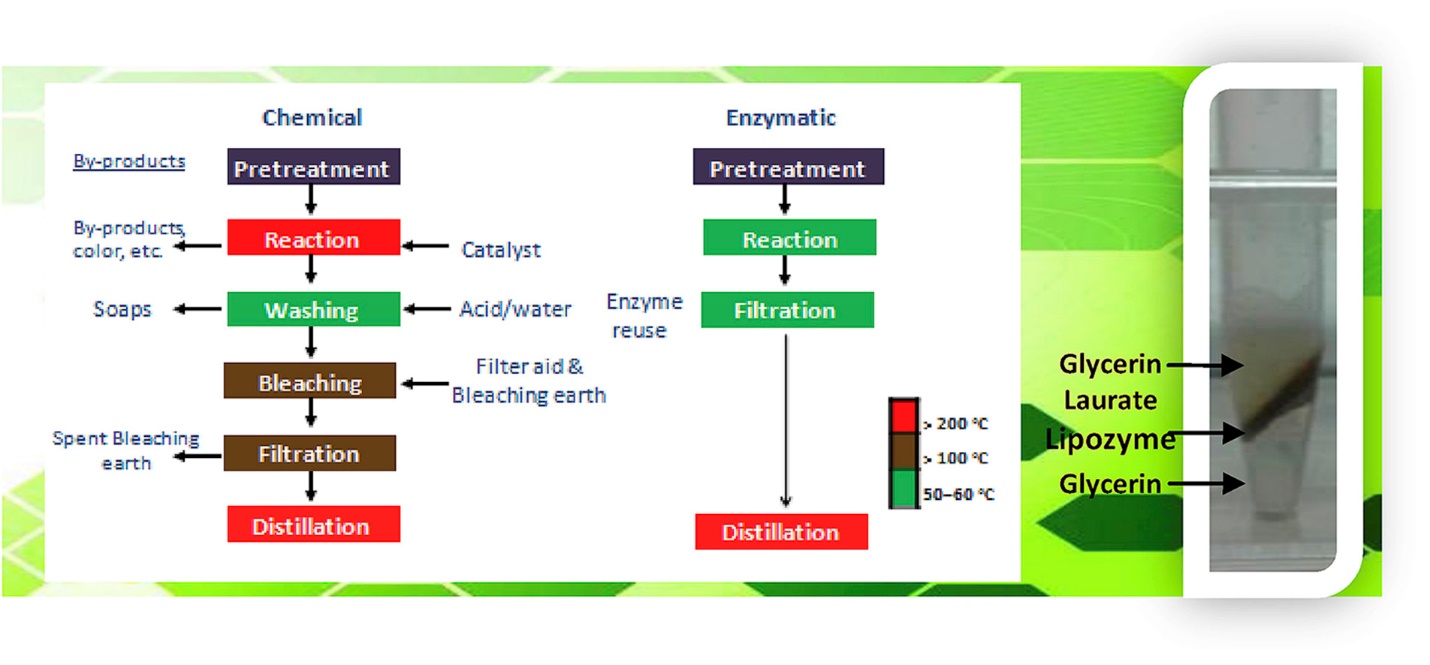
Graphical design of adjuvant production [18].

The enzymatic method used in the synthesis process had several advantages over the conventional chemical method, including reduced energy and water requirements. In addition, the enzymatic esterification reaction is selective and resulted in higher monoglyceride yields [19,20].
