# Supplementary material for: Efficacy and durability of bovine virus diarrhea (BVD) virus killed vaccine adjuvanted with monolaurin
Source: PLoS One. 2022 Jul 14;17(7):e0269031. doi: 10.1371/journal.pone.0269031 (PMC9282602; doi:10.1371/journal.pone.0269031)
Supplement: Attachment — Submitted filename: Rebuttal letter final_PONE-D-21-37629.docx [file pone.0269031.s004.docx]

**Dear Editor**

I am writing this rebuttal letter in response to reviewers' comments on this manuscript which was previously refused under submission number PONE-D-21-00023.

We appreciate your efforts on the earlier version of this manuscript. However, we are pleased to reconsider the manuscript after making most of the corrections requested by previous reviewers.

A copy of the manuscript has been attached with red markings on most of the corrections recommended by the reviewers.

**Point-by-points response to the comments:**

**Comments by Paulo Lee Ho, Academic Editor:**

1) The manuscript requires many grammatical and style edits. In its present form, it is very hard for a proper understanding. Please numerate the lines and pages to facilitate the comments;

The manuscript was totally reformed and revised from the point of linguistics by a native English language colleague.

2) There a great number of missing information, the commercial vaccine used in this study, the number of animals/groups, details on vaccine preparation.

All information regarding the commercial vaccine (Pneumo-4), number and groups of animals, and vaccine preparation have been added as required by the context of the research in **6-3. Potency test** and enough to clarify the methods used.

3) It is recommended to perform a vaccination-challenge experiment to validate and correlate the serum neutralization data to protect against the BVDV challenge infection;

Unfortunately, we were unable to perform the challenge trial. The COVID-19 pandemic has put a lot of restrictions, especially in the case of challenge trials. However, it has been kept in mind that when the opportunity is available, the challenge experiment will be our priority.

4) Please, see the comments raised by both reviewers.

Here in this file we have added the responses to the review of previous reviewers.

**Reviewer #1**

General Comments:

This is a paper providing information on the use of adjuvants with an inactivated BVDV vaccine. The paper needs extensive grammatical and style edits- a native English speaker should provide additional edits. Page and line numbers need to be added to provide specific edits in the document.

The manuscript was totally reformed and revised from the point of linguistics especially by a native English language colleague. However, we agree to run a grammatical and style edits if the journal recommends this step. The line numbers were added to submitted version.

Specific Comments:

The Ethics Statement is missing the approval-what did the Research Ethics Committee approve.

The ethical statement was added to the manuscript.

Capitalization is incorrect in the corresponding addresses and corresponding author. Where is October University?

Authorship information was corrected in terms of linguistics and full address. October University is located in 6^th^ of October City, Giza, Egypt.

Abstract needs to be rewritten- it is poorly written- it needs to be in the past tense. What kind of animals and how many animals per group needs to be provided?

The abstract was reformed and all pertinent information was added regarding the animal species and numbers.

Introduction:

With BVDV the correct term in immune dysfunction and not immune suppression as BVDV can also stimulate proinflammatory responses.

The description of immune status was corrected to immune dysfunction. However, some of previous researches described the effect of the BVDV on the immune system in animals by its suppression. We may consider the dysfunction is a sequel or an end result to the suppression.

Materials and Methods:

The first section should be entitled “viruses and cells”. Were the cells free of BVDV? It is impossible to tell as written.

The first section (Viruses and Cells) has been renamed. The cell line used in the experiment is periodically tested at the Research Institute (VSVRI) where there is expanded production of multiple commercially available vaccines in Egypt and Africa. In the sterility section, we tested the prepared vaccines and confirmed to be free of the non-cytopathic BVDV strain whereby the cell line is thus also free.

It is impossible follow the flow of this experimental design. The calf group sizes are unclear and the total number animals used in the safety and efficacy unclear.

The type and numbers of the animals were mentioned in details. Twelve native breed male calves aged 6 months were used to evaluate the potency of different vaccine formulas. In potency test, 15 calves were used and divided into 5 groups each 3 animals. In safety, 10 albino Swiss mice and 6 male calves were used.

**Reviewer #2**

Comments:

1. They mention a •**commercial vaccine** but didn’t disclose the name of the company. Also, the killed vaccines usually **•require two injections** to obtain a reasonable neutralizing-antibody titer. It is not clear whether they followed the proper instructions for the unnamed commercial vaccine. Although the experimental vaccine did induce slightly better neutralizing- antibody titers than the commercial vaccine, the authors need to perform a **•vaccination-challenge** experiment to validate and correlate their serum neutralization data to protect against the BVDV challenge infection.

- The commercial vaccine is the Pneumo-4 which is a combine killed vaccine against four respiratory viruses BVDV one of them. The vaccine is produced in the research institute (VSVRI) and an extensive research and field trials were done on it.
- Already, we missed the dosing schedule for the vaccines used and added this information in section **6-3 Potency test**.
- Unfortunately, we were unable to perform the challenge trial. The COVID-19 pandemic has put a lot of restrictions, especially in the case of challenge trials. However, it has been kept in mind that when the opportunity is available, the challenge experiment will be our priority.

2. The authors did not elaborate on their vaccine preparation in detail. Notably, they don’t mention their virus titer before virus inactivation or determine the inactivated protein concentration in their vaccine preparation? A Western blot showing the immunoblotting reactivity/status of the inactivated BVDV E2 protein need to be documented.

The virus is inactivated without separation of any of its proteins. In section **1-Viruses and cells** we mentioned that a local Egyptian strain of Bovine viral diarrhea virus genotype 1 (BVD-1) (NADL strain) (10^6.5^ TICD_50_) was supplied. The institute adopts SNT and ELISA in evaluating prepared vaccines as these methods are considered standard tests in addition to being low cost.

3. Instead of NI they should show the actual neutralizing antibody titers. Fig. 1 shows only the mean NI titer and not the standard deviation of the vaccinated animals' data set (error bars).

In new reviewing process, the data will be available if the reviewers ask to add it to the figures and/or tables.
