# Supplementary material for: Efficacy and durability of bovine virus diarrhea (BVD) virus killed vaccine adjuvanted with monolaurin
Source: PLoS One. 2022 Jul 14;17(7):e0269031. doi: 10.1371/journal.pone.0269031 (PMC9282602; doi:10.1371/journal.pone.0269031)
Supplement: Attachment — Submitted filename: Response to Reviewers.docx [file pone.0269031.s005.docx]

Dear Dr. Paulo Lee Ho

Academic Editor

PLOS ONE

I am writing regarding manuscript PONE-D-21-37629 “Efficacy and durability of Bovine Virus Diarrhea (BVD) virus killed vaccine adjuvanted with monolaurin” which was submitted in your leading journal PLOS ONE.

While we are optimistic that our manuscript reached this stage of the review, we assure that we will do the corrections and recommendations in line with the reviewers' comments, and hope to see the manuscript is published soon.

In this letter, we will respond to the reviewers' comments point by point, and will also put the required corrections in a separate file according to your request.

**Journal Requirements:**

1. Please ensure that your manuscript meets PLOS ONE's style requirements, including those for file naming.

We have revised the manuscript in accordance with the style mentioned in websites stated in the email.

2. In your Methods, please include full details of animal care and housing.

We included full details of animals care and housing.

3. Data Availability statement,

We attached a supplementary file describing adjuvant raw preparation and its production procedures.

4. Please amend either the title on the online submission form (via Edit Submission) or the title in the manuscript so that they are identical.

The correct title is:

Efficacy and durability of bovine virus diarrhea (BVD) virus killed vaccine adjuvanted with monolaurin

Short title:

Bovine virus diarrhea (BVD) virus killed vaccine adjuvanted with monolaurin

Also, the virus name is corrected in the Keywords

5. Figures files

We uploaded the figures to the Preflight Analysis and Conversion Engine (PACE) tool per your request.

**Reviewers' comments**

**Reviewer #1:**

This is a greatly improved manuscript. The authors have made the necessary corrections throughout the manuscript. The English in particular was greatly improved making the manuscript much more readable.

Thank you for inspiring comment.

**Reviewer #2:**

1-The manuscript needs grammatical revision for a proper understanding. English proof reading should be performed.

The manuscript was totally reformed and revised by [www.enago.com](http://www.enago.com) | Enago – English Editing. The certificate is included.

2-The experiment is not described in a way which allows a full understanding of a few key aspects:

*is the commercial vaccine used according to the SPC?

The commercial vaccine used in this experiment is a locally prepared vaccine named Pneumo-4 which is adjuvanted with aluminum hydroxide gel. This vaccine contains other three local isolates (BHV-1, BPIV-3, and BRSV) plus BVDV. It is considered one of the registered vaccines against the four agents by the Egyptian authorities. As I understood, *SPC* stands for *summary of product characteristics* and I believe that the manufacturing and application of the commercial vaccine were done in a close adherence to the European SPC. The rules are in terms of separate production of each agent, manufacturing QC/QA, injection route, site of administration, and poster dose, etc.

*What about the groups, how animals were assigned to each group and which criteria were used to know if the groups were equivalent?

The animals used in the experiment were selected to be equivalent in age, weight and breed. The plan was explained in detail under title **6-3 Potency test**. Antibodies level of calves were examined by SNT against the four viruses and proofed their freedom of significant titers. Also, we confirmed and took in consideration that animals were not vaccinated before against any of agents used in the experiment. Calves were divided into 5 groups as follows, control negative non vaccinated, three groups vaccinated with the BVDV vaccine adjuvanted with monolaurin 0.5%,1%, 2%. Finally, a fourth group was vaccinated with the commercial vaccine.

3-Do the other antigens in the commercial vaccine have a role in the immune response of the treated animals? In other words, is it correct to compare a monovalent vaccine with a multivalent one to assess the experimental adjuvant?

The concept of polyvalent vaccines is considered the most common in the Egyptian veterinary market. This comes from the endemicity of various threats that can affect livestock on a large scale. In this experiment, we mainly aim to examine the effect of monolaurin as an adjuvant and we have preferred to use one antigen to reduce other effects. The team plans to use the new adjuvant with the polyvalent vaccine in the near future.

We will add this note in the context of the manuscript.

4-A commercial ELISA has been used to assess the seroconversion, but very little is known about this kit and in particular its modifications, a detailed description of the modifications and the reasons why this test has been chosen is needed

We will add this detail through the manuscript regarding the test.

5-SNT technique is used to assess calves before the experiment, but nothing is mentioned about this SNT method. Moreover, what has been done to exclude animals were PI or had other immunosuppressive conditions?

We will add this detail through the manuscript regarding the test.

Regarding the PI condition and its existence, we surveyed animals against both BVDV antibodies and antigen itself. All animals were proved negative for both. We mentioned this information under **3.2. Calves**. We will add more details regarding antigen detection.

6-What has been done to exclude BVDV circulation during the study? This could also affect the results.

We agree with this view that BVDV can metastasize again during the trial. We isolated all animals used in the experiment in an isolation unit dedicated to the experimental evaluation of the produced vaccines. This isolation unit is located at the Veterinary Serum and Vaccine Research Institute (VSVRI). We will add this note in the context of the manuscript.
